# Integrative proteomics and metabolomics analysis of the mechanism of pancreatic β-cell dysfunction in aged mice

**DOI:** 10.3389/fendo.2025.1723927

**Published:** 2026-01-02

**Authors:** Fenghui Pan, Long Wang, Xuan He, Can Rong, Yun Hu

**Affiliations:** 1 Department of Geriatrics, Nanjing Drum Tower Hospital, Nanjing, Jiangsu, China; 2 Division of Geriatrics, The Third Affiliated Hospital of Soochow University, Changzhou, Jiangsu, China; 3 Department of Medicine, Jiangsu Health Vocational College, Nanjing, Jiangsu, China

**Keywords:** aging, diabetes, metabolomics, pancreatic dysfunction, proteomics

## Abstract

**Objectives:**

The prevalence of type 2 diabetes mellitus has increased worldwide and is higher among older individuals. Exploring the mechanisms underlying pancreatic β-cell dysfunction may help elucidate the pathogenesis of age-related diabetes.

**Methods:**

Islet function-related parameters were measured in four young and four aged mice. Endogenous proteins and metabolites in the pancreas were detected using liquid chromatography-tandem mass spectrometry (LC-MS/MS)-based proteomics and metabolomics, and integrated data analysis was performed.

**Results:**

Compared with young mice, aged mice presented higher fasting blood glucose levels and insulin resistance index (according to the homeostatic model assessment for insulin resistance, HOMA-IR), whereas that from the homeostasis model assessment of β-cell function (HOMA-β) significantly decreased. A total of 3,795 proteins were quantified, 57 of which were upregulated and 50 were downregulated in aged mice. Moreover, 46 metabolites were significantly upregulated and 19 were downregulated in aged mice. Integrated proteomic and metabolomic analyses revealed six significant pathways implicated in these changes, including arginine biosynthesis and the pentose phosphate pathway. By integrating comprehensive multi-omics data, the arginine biosynthesis-related metabolites aspartate and glutamine were found to be associated with the aging phenotype and islet function.

**Conclusion:**

These findings suggest that concurrent endogenous protein and metabolite disturbances occur in the pancreas of aged mice, and metabolite aspartate and glutamine may serve as potential biomarkers and therapeutic targets for aging-related pancreatic dysfunction.

## 1 Introduction

Diabetes mellitus is a chronic metabolic disorder characterized primarily by increased blood glucose levels that can trigger damage and impair the function of various organs, posing a significant threat to the well-being and quality of life of affected individuals (1). The incidence of diabetes has increased, with China leading the global ranking (2). Type 2 diabetes mellitus (T2DM) typically affects older individuals, with a prevalence rate among those aged 60 years and above approximately reaching a staggering 25%, which surpasses the general adult prevalence rate of approximately 13% in China (3). An epidemiological investigation revealed a gradual increase in the incidence of diabetes with advancing age, from 28.8% among those aged 60–69 years to 31.8%% in over 70 age population (4). Aged individuals with diabetes have a heightened risk of cardiovascular diseases, resulting in significant increases in healthcare, caregiving, and societal costs associated with the disease and concomitant cardiovascular conditions (5). Therefore, therapeutic interventions to mitigate age-related disruption of glucose metabolism are urgently required.

Conventional theories posit that insulin resistance is the primary driver of glucose homeostasis disturbances in the elderly population (6). Recent studies have underscored the crucial role of diminished islet function in the pathophysiology of T2DM in this patient subpopulation (7). Studies assessing the impact of aging on the pancreas have attracted increasing attention from research institutions and funding agencies (8). Consequently, exploring the impairment of pancreatic β-cell function has the potential to elucidate the pathogenesis of age-related diabetes and provide novel targets for the prevention and treatment of T2DM, particularly in the aging population.

Biological processes exhibit intricate and holistic characteristics, and single-omics data alone cannot systematically reveal the complex mechanisms governing disease onset and progression. Proteomics, which focuses on the composition and alterations of protein levels within cells, tissues, or organisms (9), and metabolomics, which scrutinizes variations in all metabolites within biological systems while seeking relationships with physiological or pathological changes, represent the omics that are most proximate to the phenotype (10, 11). Integrated proteomic and metabolomic analyses have the potential to identify the underlying causal mechanisms connecting genotypes to phenotypes, facilitating the exploration of biological questions from both causal and consequential perspectives and promoting mutual validation. By utilizing extensive datasets to identify key proteins, metabolites, and metabolic pathways, these approaches can bolster the prospects for in-depth research and applications in the regulation of biological systems (12).

**Figure 1:**
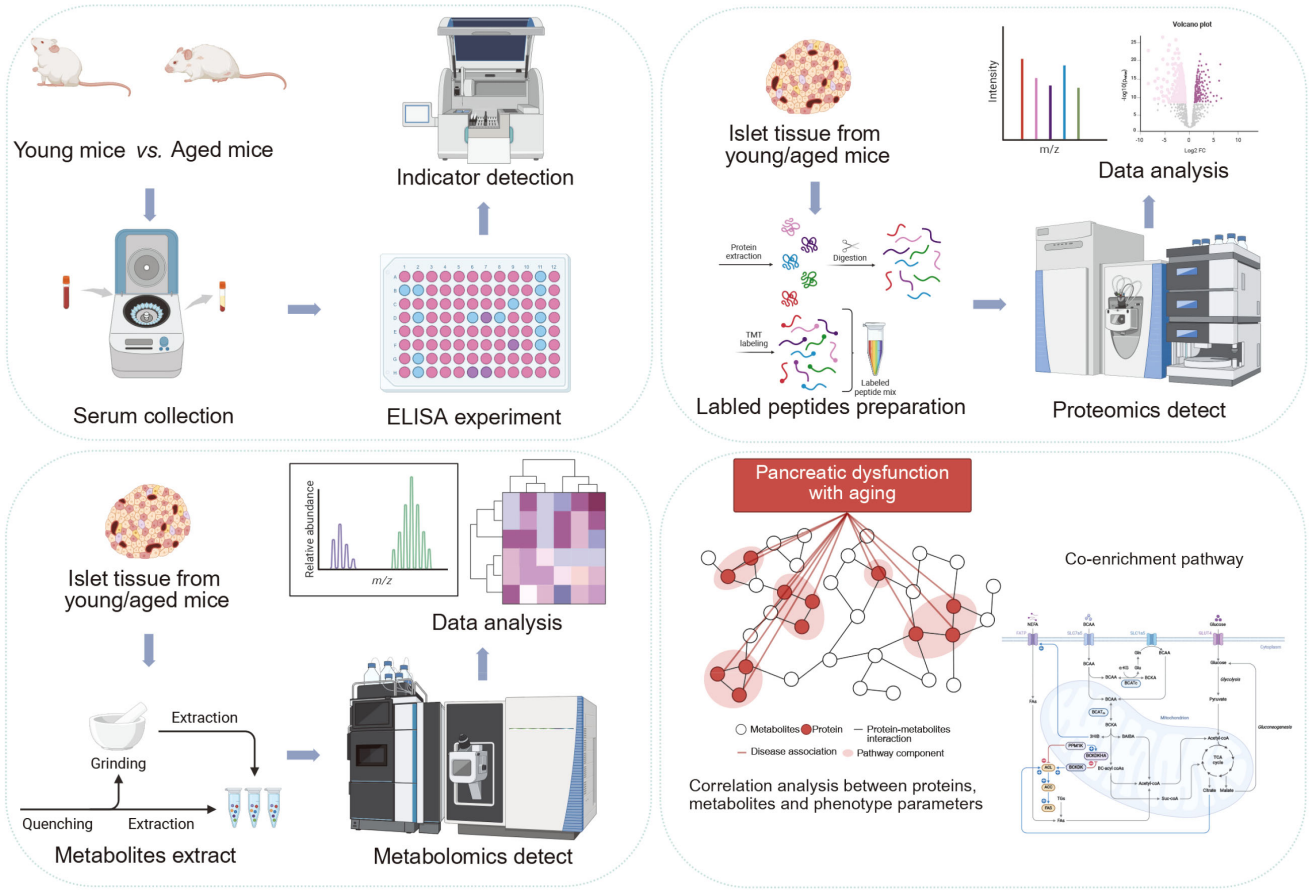
Schematic workflow of the integrated proteomic and metabolomic strategy used to characterize the mechanism of aging-induced pancreatic dysfunction in mice. First, samples from young and aged mice were collected, and body weight, blood glucose and islet function indicators were measured and compared between them. Second, proteomic profiling of the pancreas via mass spectrometry-based untargeted proteomics analysis was performed. The metabolic profile of the pancreas was subsequently analyzed via mass spectrometry-based untargeted metabonomics analysis. Finally, integrated proteomic and metabolomic analyses were used to identify key pathways associated to aging-related pancreatic dysfunction.

In this study, we comprehensively analyzed the endogenous proteins and metabolites in the pancreas of young and aged mice (**Figure 1**). We analyzed the fundamental characteristics of those that were differentially expressed and described their components, molecular functions, biological processes, and metabolic pathways through an integrated analysis. This study aimed to elucidate the relationship between age-related alterations in pancreatic proteins and metabolites, and decreased pancreatic islet function in mice, with the ultimate goal of providing novel avenues for the treatment of T2DM, particularly in aging patients.

## 2 Materials and methods

### 2.1 Animal experiments

Four young C57BL/6 mice aged 3–6 months and four aged (18–21 months) mice from the same strain were used in this study (13). The mice were housed in an SPF-grade animal rearing center at the Nanjing Drum Tower Hospital, at a temperature of 23 ± 2°C and a relative humidity of 50%–60%. The animals were subjected to a 12-hour light-dark cycle (lights on 08:00-20:00, lights off 20:00-08:00) and provided *ad libitum* access to standard rodent chow and water. After one week of acclimatization, an intraperitoneal glucose tolerance test (IPGTT) and an intraperitoneal insulin tolerance test (IPITT) were performed. The methods were as follows: after a fasting period lasting 16 or 4 hours, the mice were intraperitoneally injected with insulin (0.75 U/kg) or glucose (2.0 g/kg), respectively. Blood glucose levels were measured before and at 30, 60, 90, and 120 min after insulin or glucose injection. At 48-hour intervals after the IPITT, the mice were fasted overnight without water restriction for 12 h. We measured the fasting body weight and collected tail vein blood samples to determine fasting blood glucose (FBG) levels. We subsequently enucleated the eyeballs of the mice to obtain blood samples, which were coagulated at 4°C for 2 hours. After the blood samples were centrifuged, the obtained serum was stored at -20°C for subsequent analyses. Fasting serum insulin (FINS) and fasting C-peptide (FCP) levels were measured using enzyme-linked immunosorbent assay (ELISA) kits (Millipore, Billerica, MA, USA). We calculated the homeostasis model assessment-estimated insulin resistance (HOMA-IR) as follows: HOMA-IR = FBG × FINS/22.5, where the units for FBG were mmol/L and those for FINS were IU/mL. The value for the homeostasis model assessment of β-cells (HOMA-β) was calculated using the following formula: HOMA-β = 20 × FINS/(FBG-3.5). Prior to pancreatic tissue collection, mice were deeply anesthetized and subjected to transcardial perfusion for systemic blood clearance. Ice-cold PBS was perfused through the left ventricle with simultaneous incision of the right atrium to allow outflow. Perfusion was maintained at a constant rate until the effluent became clear and visceral tissues showed visible blanching, confirming effective blood removal. The pancreas was then rapidly dissected, gently rinsed with ice-cold PBS, and immediately snap-frozen in liquid nitrogen and subsequently stored at -80°C.

### 2.2 TMT labeling

To extract proteins from pancreatic tissue, RIPA lysis buffer was used on samples weighing 100 mg, after which the protein concentration was measured using a BCA protein assay kit (Thermo Pierce, USA). TMT-10 plex isobaric label reagent (Thermo Pierce, USA) was used to label the samples according to the manufacturer’s recommendations. Briefly, 100 mM triethyl ammonium bicarbonate (TEAB) buffer was added to 100 μg of protein. Afterward, 5 μL of 375 mM iodoacetamide was added, and the mixture was incubated at room temperature for 30 min in dark conditions. Next, 5 μL of 200 mM tris(2-carboxyethyl) phosphine (TCEP) (Sigma, USA) was added, and the samples were incubated at 55°C for 1 h. The proteins were then precipitated with acetone chilled to -20°C. Next, 2.5 μg of trypsin (Sigma) was added to digest proteins, the samples were incubated overnight at 37°C, and the resulting digested peptides were resuspended in 100 μL of 100 mM TEAB. TMT-10 plex isobaric label reagent was added to label the peptides, followed by incubation at room temperature for 1 h, and 5% hydroxylamine was used to stop the reaction. Finally, the eight samples with labeled peptides were combined for further fractionation.

### 2.3 High-pH fractionation

Before fractionating the labeled peptides, the samples were desiccated and reconstituted in a solvent containing 2% acetonitrile at pH 10. The labeled peptides were fractionated using an Xbridge PST C18 column (130 Å, 5 μm, 250 × 4.6 mm; Waters, USA). The reverse-phase liquid chromatography (RPLC) gradient for solvent B (90% acetonitrile, pH 10) changed from 5% to 95% over a period of 40 min. The flow rate was maintained at 1 mL/min, and the column temperature was equal to that of room temperature. Fractions were collected every minute, resulting in 40 fractions of equal volume. The fractions were then vacuum-dried and stored at -80°C for subsequent analysis using liquid chromatography-tandem mass spectrometry (LC-MS/MS).

### 2.4 LC-MS/MS analysis

The LC-MS/MS analysis was performed using a Q Exactive HF-X Orbitrap System (Thermo Scientific, Waltham, MA, USA). The samples were dissolved in a solution containing 0.1% formic acid and 4% acetonitrile (TEDIA, USA). Peptides were separated using an Acclaim PepMap 100 precolumn (75 μm × 2 mm) and eluted via an Acclaim PepMap RSLC C18 analytical column (75 μm × 15 cm) (Thermo Scientific). The flow rate was 300 nL/min with a gradient of solvent B from 3% to 30% (90% acetonitrile with 0.1% formic acid) for 43 min, followed by > 30% for 1 min. The Q Exactive HF Orbitrap System employs a data-dependent acquisition mode with a specified full scan (m/z range from 375 to 1500, nominal resolution of 70000), followed by MS/MS scans of the 20 most intense fragments in each cycle (m/z range from 200 to 2000, nominal resolution of 35000). Tandem mass spectra were generated by high-energy collision dissociation (HCD) with a dynamic exclusion period of 18 s.

### 2.5 Data analysis and TMT quantification

For data analysis, a database search against the RefSeq mouse protein sequence database was performed using the Proteomics Discoverer software (version 1.4; Thermo Scientific). The search criteria included a mass tolerance of 15 ppm for precursor ions and 20 mmu for fragment ions. Trypsin was selected as the digestion enzyme, allowing for up to two missed cleavages. The fixed modifications included cysteine carbamidomethylation and TMT modifications at the N-terminus and lysine residues, whereas methionine oxidation was treated as a variable modification. The results were filtered using the following settings: only highly confident peptides with a global false discovery rate (FDR) <1% based on the target-decoy approach were included in the results. TMT quantification features the most accurate centroid method with a 20 ppm integration window and uses unique peptides for protein quantification. Missing values were imputed automatically, with less than 25% of missing data points replaced by values from a normal distribution. This distribution is typically downshifted and reduced to simulate low-abundance signals, thereby enabling a more robust statistical analysis. This distribution is typically shifted downward and narrowed to simulate low-abundance signals, allowing for a more robust downstream statistical analysis. Subsequently, the data were normalized by median centering to align the median protein abundance across all TMT channels, correcting for systematic technical variance.

### 2.6 Metabolite extraction and identification from pancreatic tissue

To extract metabolites from pancreatic tissue, we lysed 50 mg of the sample and added four volumes of chilled methanol. For protein precipitation, the tube was vortexed and stored at -80 °C for 8 h. The mixture was centrifuged at 14,000 rpm for 10 min at 4 °C. The supernatant was discarded, followed by evaporation to dryness, reconstitution, and analysis using an ultra-performance liquid chromatograph (UPLC) connected to a Q Exactive HF-X Orbitrap tandem mass spectrometer. Metabolite separation was achieved using reversed-phase liquid chromatography (RPLC), and the metabolites were analyzed in both positive and negative electrospray ionization (ESI) modes. MS/MS data were acquired in both full- and product-ion scan modes (m/z range: 67–1000). Data-dependent acquisition (DDA) was used to collect the secondary mass spectra with high sensitivity. To investigate the stability of the system, quality control (QC) samples derived from plasma aliquots were introduced every 10 samples. MS-DIAL (version 4.36) was used for the deconvolution, peak identification, alignment, and compound identification.

### 2.7 Data analysis

Statistical analyses, including Student’s t-test, Mann-Whitney U test, variance analysis, and Spearman correlation, were performed using SPSS software (version 22.0; Chicago, USA). Multivariate data analyses such as principal component analysis (PCA), orthogonal partial least-squares discriminant analysis (OPLS-DA), heatmap and volcano plot analysis were conducted using R studio software (version 4.2.1). Statistical significance was set at a *P*-value < 0.05. The O2PLS analysis was performed using the OmicsPLS package in R. Prior to modeling, the protein and metabolite abundance matrices were mean-centered to mitigate the influence of differing measurement scales. Model parameters were optimized via 10-fold cross-validation using the crossval_o2m function. We systematically evaluated combinations of 1 to 3 joint components, alongside 0 to 3 orthogonal components for the proteome and 0 to 3 for the metabolome. The final model configuration—selected based on the minimal prediction error—comprised 2 joint components, 2 proteome-specific orthogonal components, and 0 metabolome-specific orthogonal components. A scatter plot was generated to visualize both proteins and metabolites within the same joint component space, illustrating the relative distribution of the two omics datasets. Point sizes correspond to variable importance, represented by absolute loading values, with the top 20 influential variables (10 proteins and 10 metabolites) displayed. Spearman correlation analysis was used to explore the association between islet function indicators and differential metabolites or proteins, and the significant threshold of the correlation was set at r >0.9. Core metabolites with a metabolite-protein-islet function indicators degree > 25 were identified. Multi-omics networks were constructed using Cytoscape (v3.10.0) software to elucidate their interactions.

## 3 Results

### 3.1 Comparison of body weight, blood glucose, and islet function indicators

**Figure 2:**
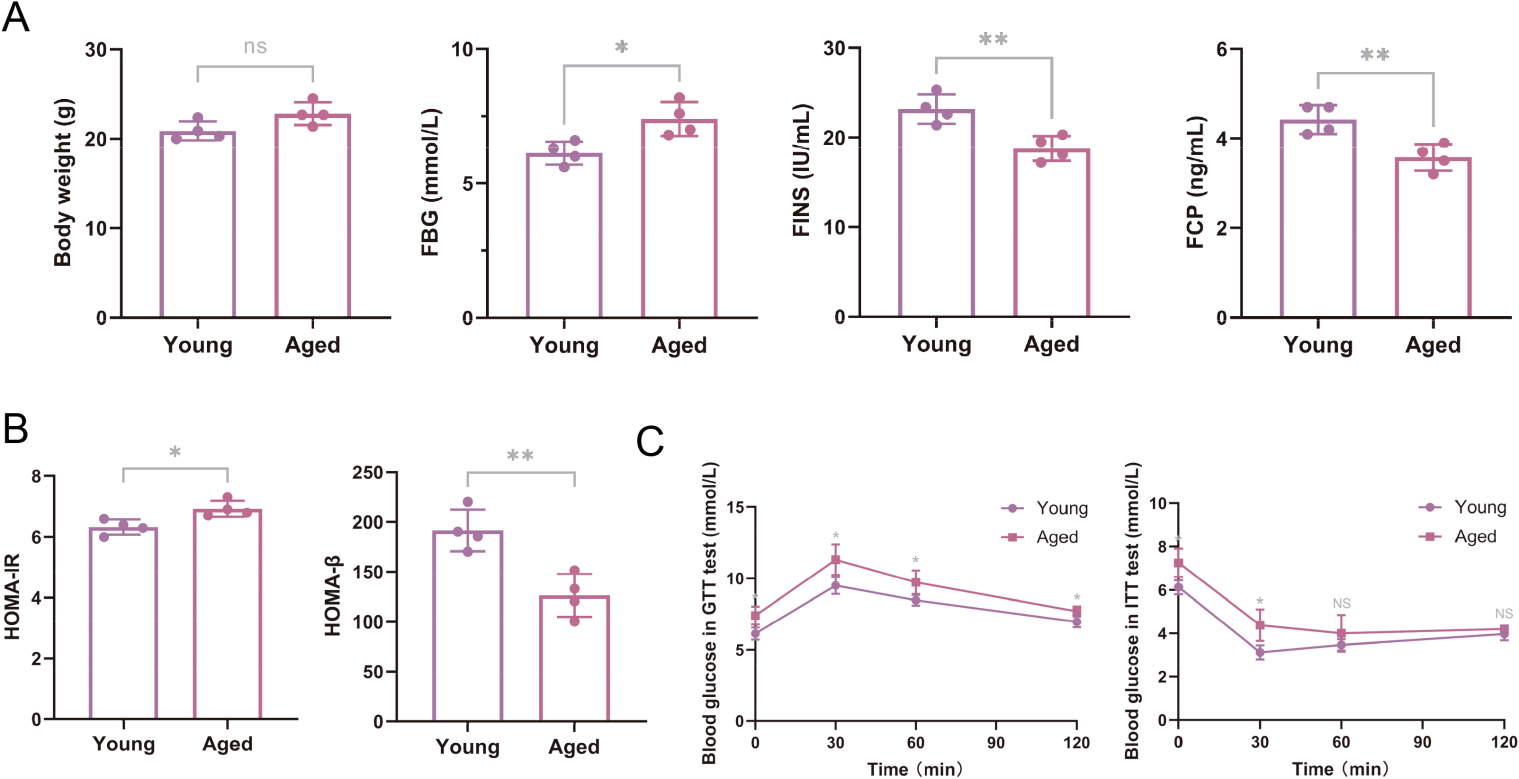
Comparison of body weight, blood glucose and islet function indicators in young and aged mice. **(A)** Body weight and fasting blood glucose (FBG), fasting insulin (FINS), and fasting C‐peptide (FCP) levels were compared between young (3–6 months old, n = 4) and aged (18–21 months old, n = 4) C57BL/6 mice. **(B)** Values for the homeostasis model assessment-estimated insulin resistance (HOMA-IR) and homeostasis model assessment of β-cells (HOMA-β) were compared. **(C)** Plasma glucose curves from the glucose tolerance test (GTT) and insulin tolerance test (ITT) were generated after the experimental subjects had undergone fasting for 16 and 4 hours, respectively. The data are presented as the means ± SDs, ^*^P < 0.05; ^**^P < 0.01.

Compared with young mice, aged mice showed no statistically significant differences in body weight (*P* > 0.05). However, FBG levels significantly increased (*P* < 0.05), whereas FINS and FCP levels significantly decreased (*P* < 0.01) (**Figure 2A**). Compared with young mice, aged mice had significantly higher values for HOMA-IR (*P* < 0.05) and lower values for HOMA-β (*P* < 0.01) (**Figure 2B**). The results of the GTT (**Figure 2C**) indicated that after intraperitoneal glucose injection, aged mice had higher blood glucose levels than young mice at various time points. The differences were statistically significant at 0, 30, 60, and 120 min (*P* < 0.05). ITT results (**Figure 2C**) revealed that after intraperitoneal insulin injection, aged mice had significantly higher blood glucose levels than young mice at various time points, with statistically significant differences at 0 and 30 min (*P* < 0.05 and *P* < 0.01, respectively).

### 3.2 Differential proteomic analysis of pancreatic tissue

**Figure 3:**
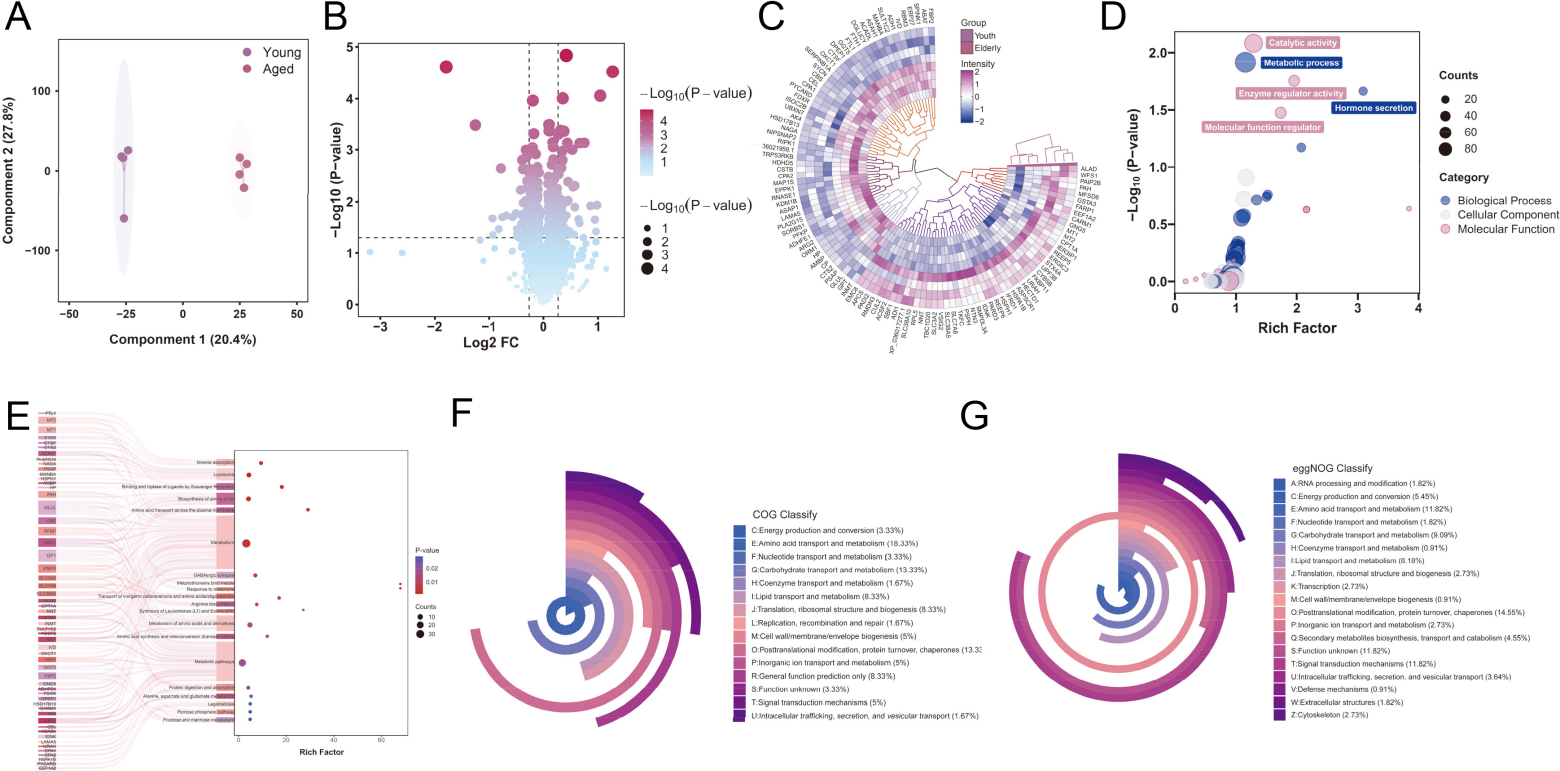
Proteomic profiling of the pancreas in young and aged mice via mass spectrometry-based untargeted proteomics analysis. **(A)** OPLS-DA plots of individual samples from young and aged mice. **(B)** Volcano plot of increased/decreased proteins. **(C)** Heatmap of relative protein abundance (fold change >= 1.2, *P* < 0.05), which revealed 57 upregulated and 50 downregulated proteins in aged mice compared with young mice. **(D)** The top Gene Ontology (GO) analysis terms in the biological process, cellular component and molecular function categories associated to the dysregulated proteins. **(E)** Differentially expressed proteins were mapped to canonical pathways using the Kyoto Encyclopedia of Genes and Genomes tool. Clusters of orthologous groups (COG) classification **(F)** and evolutionary genealogy of genes: unsupervised orthologous groups (eggNOG) classification **(G)** analysis of differentially expressed proteins. OPLS-DA, orthogonal partial least-squares discriminant analysis.

Pancreatic tissues were subjected to TMT-labeled proteomic analyses. Raw mass spectrometry data were processed using the Proteomics Discoverer software and searched against the corresponding protein database for each species. The search results were filtered, and highly reliable peptides with less than 1% false discovery rate (FDR) were selected for qualitative protein identification. Specific peptides were chosen for relative quantitative analysis of different samples. The analysis identified 3,928 proteins and quantified 3,795 of them, with 24,971 peptide species and 66,382 peptide occurrences. Statistical analysis of peptide sequence length (**Supplementary Figure S1A**) and protein molecular weight and coverage were measured (**Supplementary Figure S1B**). OPLS-DA score plots showed clear boundaries between the pancreatic tissue from young and aged mice, indicating significant changes in protein composition (**Figure 3A**). Volcano plot analysis revealed 57 upregulated proteins (≥ 1.2-fold, *P* < 0.05) and 50 downregulated proteins (≤ 0.83-fold, *P* < 0.05) in aged mice compared with those in young mice (**Figure 3B**), while the top 10 upregulated and downregulated proteins are shown in the **Supplementary Table S1**. A visual representation (heatmap) of the protein expression levels indicates upregulation (purple) and downregulation (blue), with darker colors indicating higher expression (**Figure 3C**). Gene Ontology (GO) analysis, including cell components, molecular functions, biological processes (**Figure 3D**), and pathways (**Figure 3E**), was also conducted on the differentially expressed proteins. Additionally, cluster of orthologous group (COG) analysis (**Figure 3F**), evolutionary genealogy of genes, and non-supervised orthologous group (eggNOG) analysis (**Figure 3G**) revealed that differentially expressed proteins and their homologous clusters were involved in multiple pathways. The protein-protein interaction network of the differentially expressed proteins is shown in **Supplementary Figure S2A**. InterScan software was used to annotate the protein sequences for protein domain analysis, which is crucial for understanding the biological functions and evolution of proteins (**Supplementary Figure S2B**).

### 3.3 Differential metabolomic analysis of pancreatic tissue

**Figure 4:**
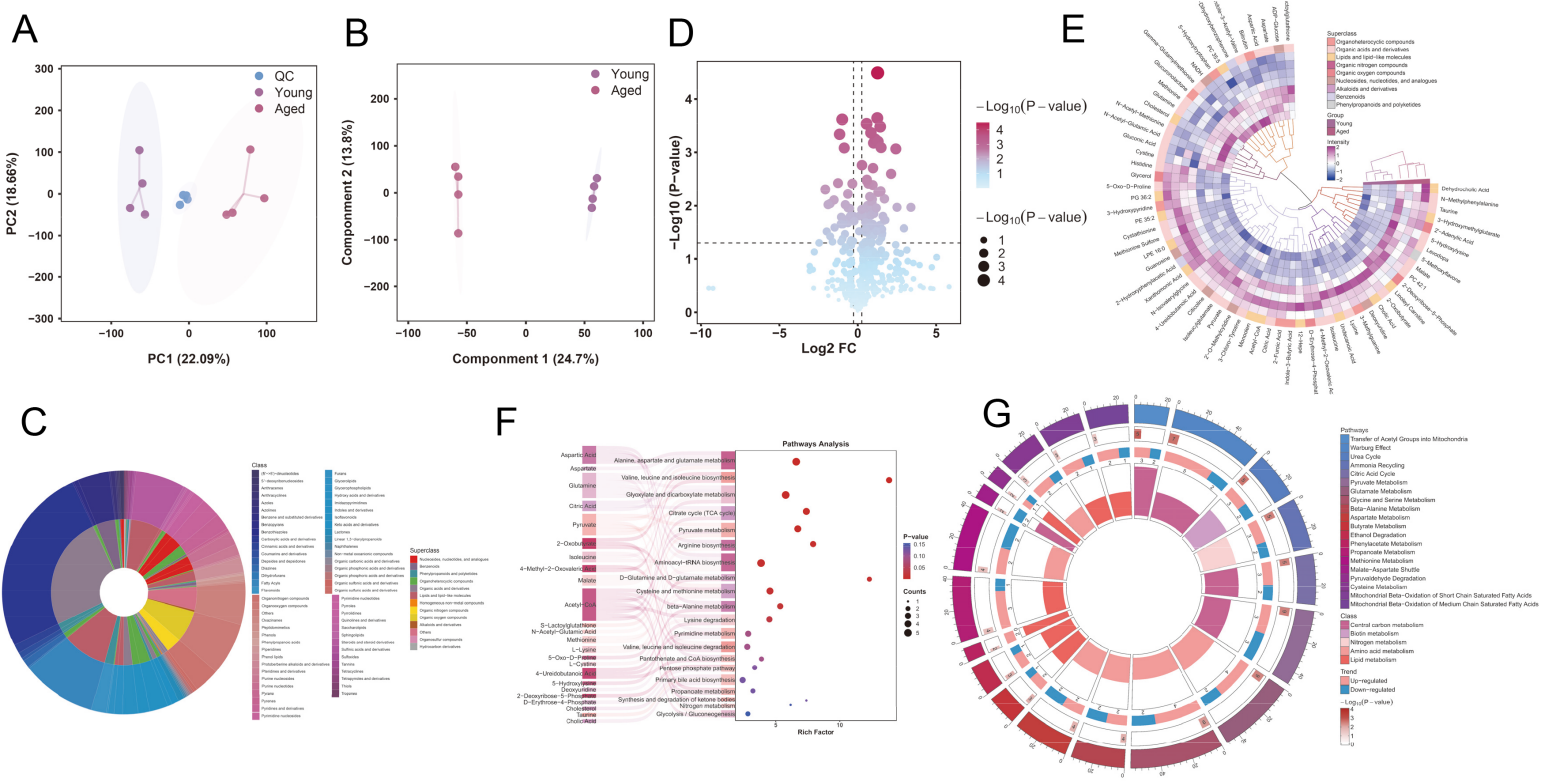
Comparison of metabolic profiles in the pancreas of young and aged mice via mass spectrometry-based untargeted metabonomics analysis. **(A)** PCA plots of individual samples from young and aged mice, and QC samples. **(B)** OPLS-DA plots of the pancreas samples from young and aged mice. **(C)** Pie chart representing the coverage of identified metabolite classes. **(D)** Volcano plot of significantly increased/decreased metabolites. **(E)** Heatmap of the relative abundance of the differentially expressed metabolites (fold change >= 1.5, *P* < 0.05), which revealed that 46 metabolites were upregulated and 19 metabolites were downregulated in aged mice compared to young mice. Differentially expressed metabolites were mapped to canonical pathways using the Kyoto Encyclopedia of Genes and Genomes tool **(F)** and metabolite set enrichment analysis **(G)**. OPLS-DA, orthogonal partial least-squares discriminant analysis; PCA, principal component analysis; QC, quality control.

We subsequently conducted a non-targeted metabolomic analysis to explore the metabolic characteristics of pancreatic tissue in aging and young mice. Overall, in all tissue samples, we discovered an excess of 20,000 metabolic features, including 11,542 in ESI-positive mode and 12,175 in ESI-negative mode. The QC sample showed tight clustering, confirming the stability and reproducibility of the analysis. Moreover, in the unsupervised analysis, the metabolite peaks in the pancreatic tissue of aging and young mice were noticeably different (**Figure 4A**). OPLS-DA score plots revealed distinct boundaries between the pancreatic tissue metabolite profiles of aging and young mice in both ESI-positive and ESI-negative modes, indicating significant age-related alterations in the metabolic landscape (**Figure 4B**). After incorporating the differentially abundant metabolite features from a common metabolite library, we conducted secondary identification and confirmed the results for 501 metabolites across multiple categories, including the primary ion peak at 3956 in MS1 assigned to 358 metabolites and the fragment ion peak at 966 in MS2 assigned to 143 metabolites (**Figure 4C**). To identify high-confidence differentially abundant metabolites in aging pancreatic tissue, we distinguished differentially abundant metabolites between ESI-positive and ESI-negative ions based on the criteria of a fold change (FC) > 1.5 and a *P* value < 0.05. Additionally, we selected metabolites with variable importance in projection (VIP) values greater than 1.0, which was calculated using OPLS-DA, as significant changes for further analysis. Subsequently, we analyzed the differentially expressed metabolites using a volcano plot, which revealed that 46 metabolites were significantly upregulated and 19 metabolites were significantly downregulated in aging mice compared to those in young mice (**Figure 4D**); the top 10 upregulated and downregulated metabolites are shown in **Supplementary Table S2**. Furthermore, we visually presented the relative expression levels of the metabolites in a heat map, with purple indicating upregulation, blue indicating downregulation, and darker colors indicating higher expression levels (**Figure 4E**). To further explore the potential functions of the significantly dysregulated metabolites, we conducted Kyoto Encyclopedia of Genes and Genomes (KEGG) pathway enrichment analysis (**Figure 4F**) and metabolite set enrichment analysis (MSEA) (**Figure 4G**), revealing the involvement of the identified metabolites in multiple metabolic pathways.

### 3.4 Integrated proteomic and metabolomic analysis of pancreatic tissue

**Figure 5:**
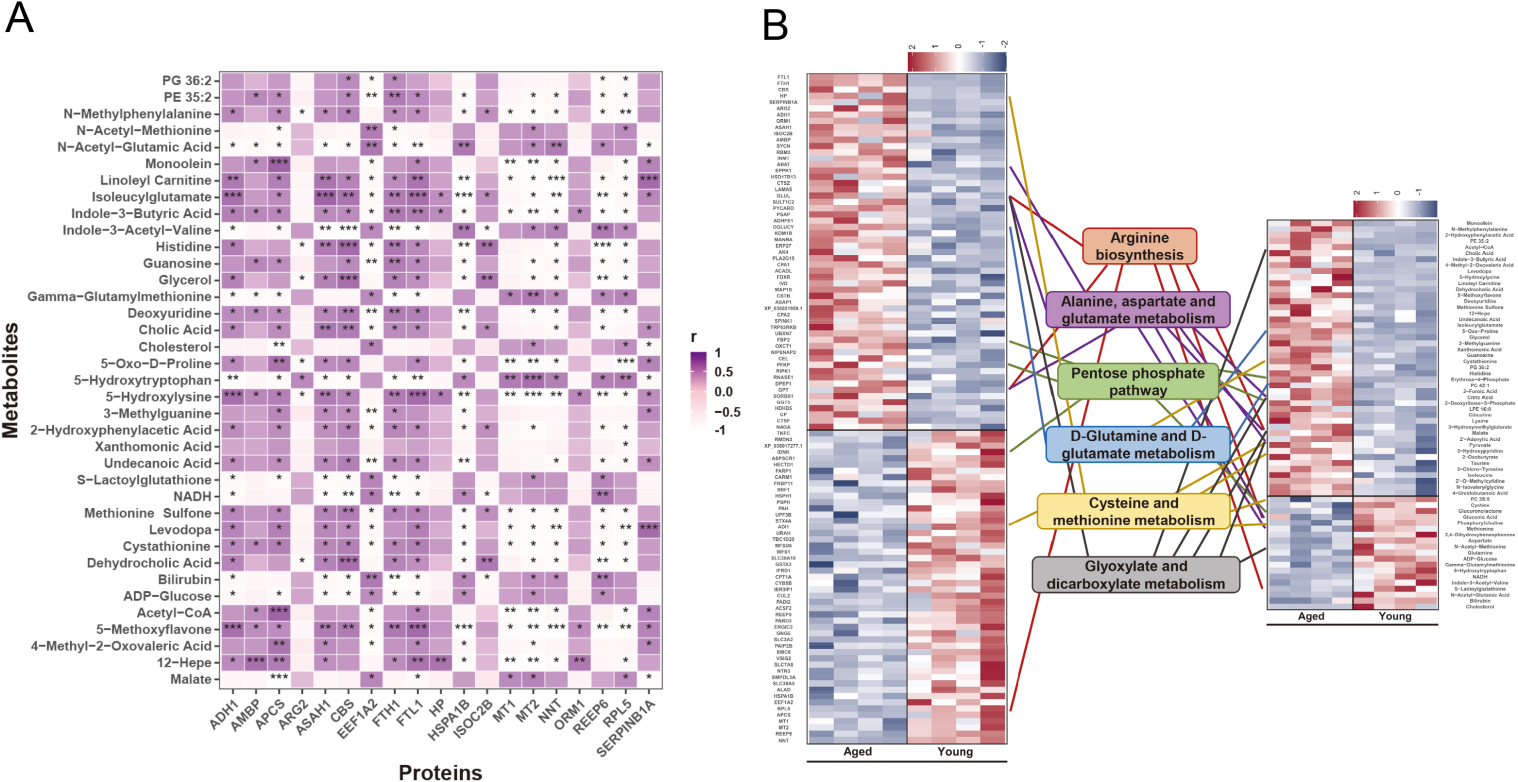
Integrated proteomic and metabolomic analysis. **(A)** Spearman correlation analysis of differentially expressed proteins and metabolites. ^*^P < 0.05; ^**^P < 0.01, ^***^P < 0.01. **(B)** Differentially expressed proteins (left side) and metabolites (right side) jointly participate in six different pathways (center).

**Figure 6:**
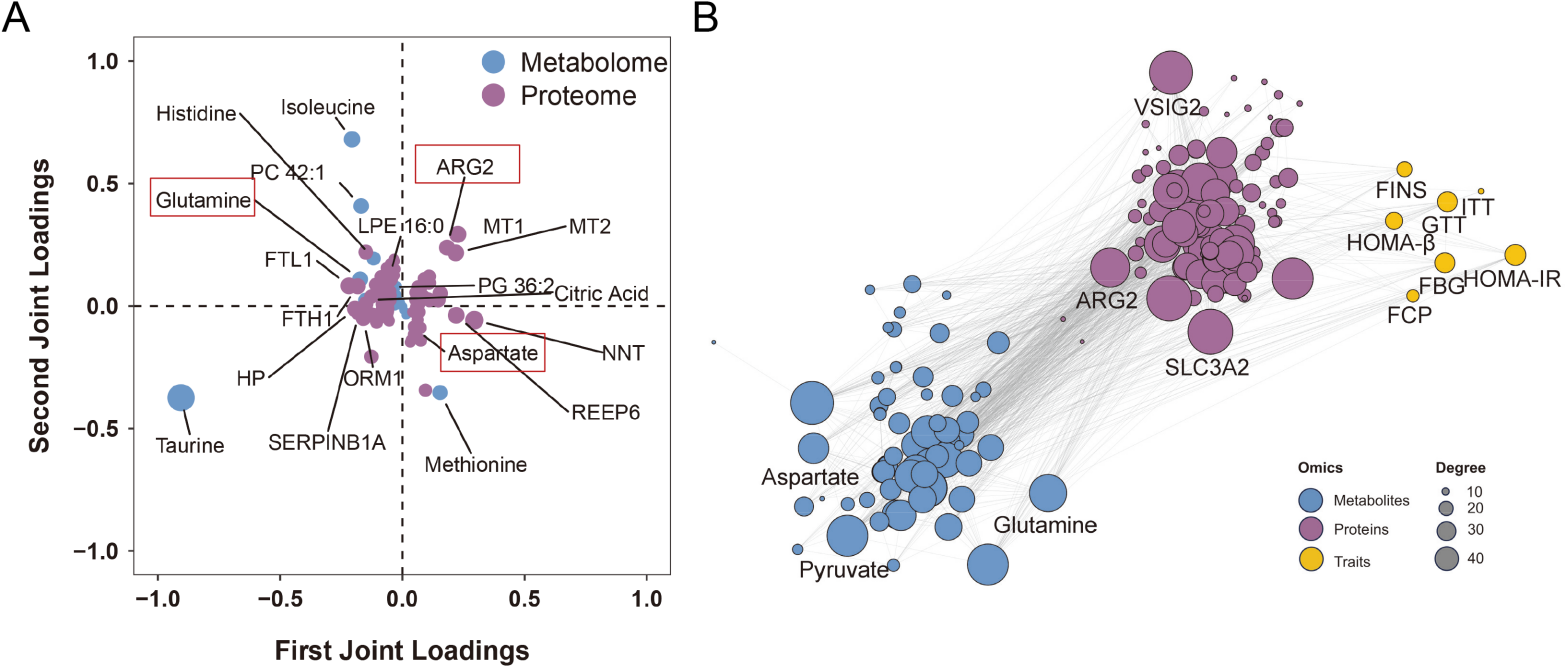
Integrated differentially expressed proteins/metabolites and islet function indicators. **(A)** Evaluation of O2PLS in proteomics and metabolomics data integration to identify key proteins or metabolites responsible for the associations. **(B)** Spearman correlation analysis of differentially expressed proteins and metabolites, and islet function indicators (including FBG, FINS, FCP, HOMA-IR, HOMA-β, GTT, and ITT). FBG, fasting blood glucose; FCP, fasting C-peptide; FINS, fasting serum insulin; GTT, glucose tolerance test; HOMA-IR, homeostasis model assessment-estimated insulin resistance; HOMA-β, homeostasis model assessment of β-cells; ITT, insulin tolerance test; O2PLS, two-way orthogonal partial least squares.

Finally, an integrated analysis of differentially expressed proteins and metabolites was conducted. The correlations between differentially expressed proteins (fold change > 1.2, *P*-value < 0.05) and metabolites (fold change > 2.0, *P*-value < 0.05) are shown in **Figure 5A**. Pathways in which differentially expressed proteins and metabolites jointly participated were identified (**Figure 5B****),** with the left side of the figure representing differentially expressed proteins, the right side representing differentially expressed metabolites, and the center depicting the pathways enriched in each case. In enrichment of integrative proteomics and metabolomics analysis, significant 6 differential pathways (FDR<0.05), including proteins and metabolites, are shown in **Supplementary Table S3**. O2PLS, a generalized form of OPLS, allows bidirectional modeling and prediction in two data matrices (14). To further explore the functional roles of key metabolites and proteins, we explored the internal connections between them while identifying the top 10 metabolites and 10 proteins with the highest correlation responsible for the aging phenotype (**Figure 6A**). Among these integrated pathways of interest, arginine biosynthesis, including the metabolic enzyme arginase 2 (ARG2), and the metabolites aspartate and glutamine, which were identified as the highest-ranking integrated pathways in our analysis, were shown to have the highest correlation (**Figure 6A**). Through correlation analysis of protein-metabolite-phenotype associations, VSIG2 and SLC3A2 proteins and the metabolite pyruvate, which had the highest degree value, were associated with islet function indicators (**Figure 6B**). More importantly, ARG2, aspartate, and glutamine were strongly associated with indicators of islet function (**Supplementary Table S4**). Afterwards, an MRM-based target metabolite aspartate and glutamine quantification method was developed, and the abundances of aspartate and glutamine in animals and humans are shown (**Supplementary Figure S3A**). Consistently, respective abundance of aspartate and glutamine in serum and tissue were detected (**Supplementary Figure S3B**).

## 4 Discussion

The incidence of T2DM significantly increases with advancing age, yet the underlying mechanisms remain unclear. Age-related insulin resistance stemming from obesity, muscle atrophy, mitochondrial dysfunction, oxidative stress, and heightened inflammation are the principal pathogenic factors of geriatric diabetes (15). However, some studies have indicated that insulin resistance alone may be insufficient to induce T2DM. With increasing age, impaired pancreatic islet β-cell function emerges as another pivotal factor contributing to anomalous glucose metabolism (6). A negative correlation between age and pancreatic β-cell function has been revealed in both basic and clinical research (16, 17). Our study revealed elevated fasting blood glucose levels, decreased fasting insulin and C-peptide levels, augmented HOMA-IR indices, and diminished HOMA-β indices in aged mice. These findings suggest concurrent insulin resistance and β-cell dysfunction in these individuals, potentially leading to synergistic disturbances in glucose metabolism.

Analyzing the correlation between the proteomics and metabolomics results obtained using these two approaches could help reveal the molecular mechanisms underlying phenotypic changes and identify key proteins, metabolites, or pathways (18). In this study, several metabolic pathways, including multiple amino acid metabolic pathways, pentose phosphate, glyoxylic acid, and dicarboxylic acid metabolic pathways, were identified through a comprehensive analysis of differentially expressed proteins and metabolites. Among these integrated pathways of interest, arginine biosynthesis, including the metabolic enzyme ARG2 and the metabolites aspartate and glutamine, was identified as the highest-ranking integrated pathway in our results. Arginine acts as both a powerful insulin secretagogue and a precursor for nitric oxide (NO), urea cycle intermediates, and polyamines, which together regulate β-cell survival, microvascular perfusion, and inflammatory stress (19). With aging, upregulated arginase—especially ARG2 reduces arginine availability for NO synthesis, leading to eNOS uncoupling, oxidative stress, and impaired vascular function, thereby undermining islet perfusion and β-cell function (20, 21). In human β-cells, mitochondrial ARG2 regulates the balance between arginine metabolism and polyamine synthesis, contributing to β-cell dysfunction in T2DM (22). Clinically, arginine perturbation is associated with risk of T2DM (23), and NO bioavailability in red blood cells declines over the course of T2DM, with plasma arginase activity rising (24). Increasing clinical evidence has shown that dietary arginine supplementation can reverse insulin resistance and alleviate T2DM (25).

Arginine biosynthesis product glutamine is essential for β cell bioenergetics: deprivation of glutamine impairs mitochondrial respiration, triggers endoplasmic reticulum stress, and diminishes insulin production and secretion, especially under lipotoxic conditions (26). Moreover, circulating glutamine levels have been reported to decline in T2DM, suggesting systemic glutamine deficiency may exacerbate β cell dysfunction over time (26). *In vitro* studies have shown that glutamate generated from glutamine can enhance the effect of calcium signaling on the amplification of glutamine-mediated insulin secretion (27). Another key metabolite, aspartate, supports mitochondrial redox balance and NADH transport via the malate-aspartate shuttle, which is critical for efficient insulin secretion (28). With aging and in diabetic states, dysregulation of this shuttle (e.g., through changes in aspartate aminotransferase or GOT1) may drive a shift toward glycolysis, as seen in senescent and diabetic β cells, impairing cellular identity and function (29). Disruption of this aspartate-dependent metabolic machinery thereby links age-associated metabolic rewiring to β cell failure and the pathogenesis of T2DM (29).

In this study, a correlation analysis of protein-metabolite-phenotype interactions was conducted, and several differentially expressed proteins and metabolites related to aging phenotype were identified. Some of these proteins and metabolites were found to be related to diabetes and insulin secretion. A clinical study revealed that the plasma levels of V-set and immunoglobulin domain-containing 2 (VSIG2) are related to glucose metabolism disorders and the occurrence of T2DM (30). Solute carrier family 3 member 2 (SLC3A2) is an important member of the solute carrier family and is involved in amino acid transport. Basic research has shown that the Zfp148 gene specifically deleted from the pancreatic β cells mouse model exhibits improved glucose tolerance and insulin secretion, and RNA-Seq and proteomic analysis of their pancreatic islets revealed changes in SLC3A2 levels. Increases in SLC3A2 and other proteins may mediate changes in the metabolism of certain amino acids, leading to increased insulin secretion (31), which is consistent with our findings of SL3CA2 downregulation and reduced insulin secretion in aged mice. Previous studies have shown that the pyruvate metabolic cycle plays an important role in controlling insulin secretion. Mitochondria use pyruvate to produce ATP, which is required for insulin secretion in β cells after glucose stimulation (32). The pyruvate cycle involved in the production of insulin secretion signals includes pyruvate/malic acid, pyruvate/citric acid, and pyruvate/isocitrate cycles (33). In addition, multiple lines of evidence have suggested that the pyruvate cycle is disrupted in animal models of islet dysfunction and T2DM (34, 35).

The present study has several limitations. First, the relatively small sample size reduces the statistical power, increases the risk of false-negative findings, and limits the ability to fully account for biological variability or confidently assess reproducibility. In addition, organizational heterogeneity arising from differences in cellular composition, metabolic state, or tissue architecture may have influenced the observed outcomes and constrained the generalizability of our conclusions. Together, these factors highlight the need for further comprehensive experiments, ideally involving larger and more diverse cohorts, as well as refined functional and mechanistic assays to gain a deeper understanding of the molecular pathways that regulate pancreatic function.

In summary, our research indicates that diminished β-cell function in aged mice is one of the reasons for impaired glucose tolerance. Using LC-MS/MS-based multiomics techniques, we analyzed the protein and metabolomic profiles of the pancreas in young and aged mice and identified numerous differentially expressed proteins and metabolites. Bioinformatics analysis suggested that these differentially expressed proteins and metabolites, aspartate and glutamine, may be involved in biological processes related to diabetes and β-cell function. This study represents only an initial exploration of pancreatic protein metabolism and its association with age-related functional decline in β-cells.

## Data Availability

The datasets presented in this study can be found in online repositories. The names of the repository/repositories and accession number(s) can be found below: http://www.proteomexchange.org/, IPX0010354000.
