# Supplementary material for: Integrative proteomics and metabolomics analysis of the mechanism of pancreatic β-cell dysfunction in aged mice
Source: Front Endocrinol (Lausanne). 2026 Jan 2;16:1723927. doi: 10.3389/fendo.2025.1723927 (PMC12807971; doi:10.3389/fendo.2025.1723927)
Supplement: Supplementary file 1 [file DataSheet1.docx]

**Integrative proteomics and metabolomics analysis of the mechanism of pancreatic β-cell dysfunction in aged mice**

Fenghui Pan^1#^, Long Wang^2#^, Xuan He^1^, Can Rong^3^, Yun Hu^1,*^

^1^Department of Geriatrics, Nanjing Drum Tower Hospital, Nanjing, Jiangsu, China.

^2^Division of Geriatrics, The Third Affiliated Hospital of Soochow University, Changzhou, Jiangsu, China

^3^Department of Medicine, Jiangsu Health Vocational College, Nanjing, Jiangsu, China

^#^These authors contributed equally to this work.

*Corresponding e-mail: Yun Hu, huyun304@njglyy.com**Supplementary Information**

**Table S1.** Top 10 differentially downregulated and upregulated proteins.

**Table S2.** Top 10 differentially downregulated and upregulated metabolites.

**Table S3.** Significant 6 differential pathways including proteins and metabolites.

**Table S4.** Core metabolites with a metabolite-protein-islet function indicators degree > 25.

**Figure S1.** Features of the quantified peptides and proteins. (A) Distribution map of identified peptide lengths. (B) Molecular weight and coverage distribution map of the identified proteins.

**Figure S2.** (A) The protein‒protein interaction network of the differentially expressed proteins. The node size corresponds to the node degree, and the color indicates the fold change, with purple representing upregulated proteins and blue representing downregulated proteins. (B) The top 30 domain enrichment analyses of the differentially expressed proteins.

**Figure S3.** (A) A typical MRM chromatogram of target metabolites. (B) Respective abundance of aspartate and glutamine in serum and tissue.

**Method S1.** Multiple reaction monitoring (MRM)-based aspartate and glutamine quantification

**
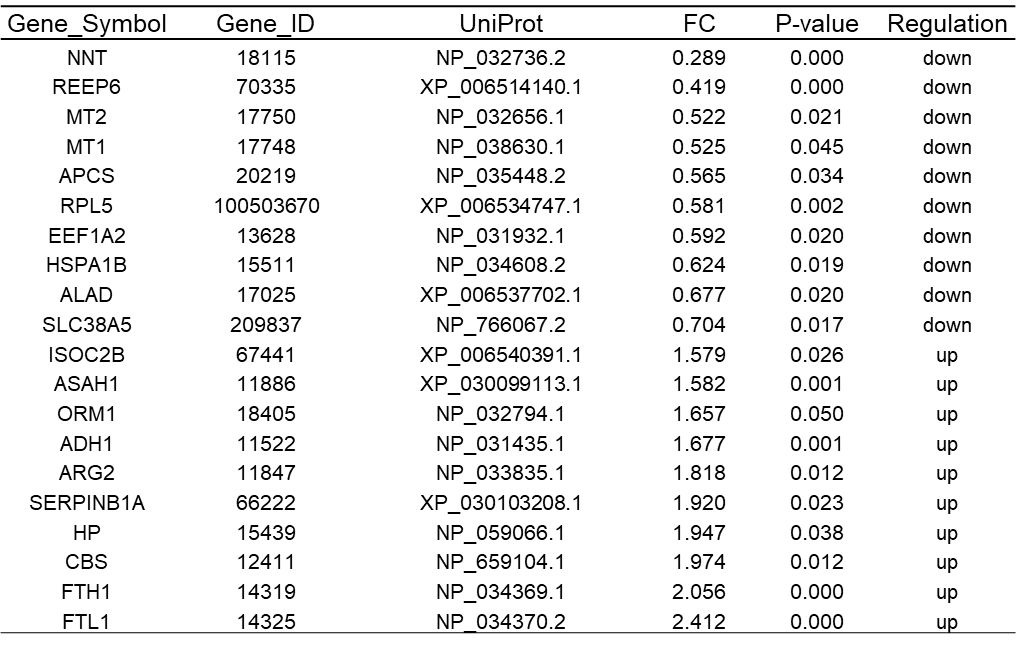
**

Table S1. Top 10 differentially downregulated and upregulated proteins.

**
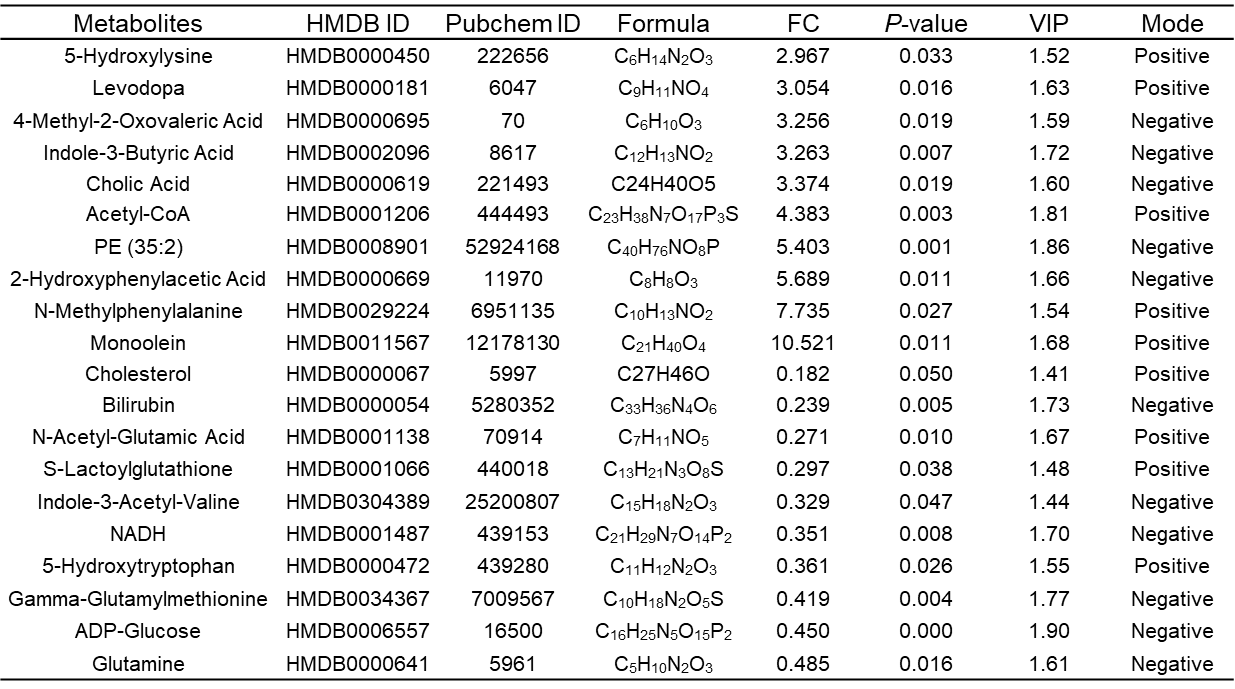
**

Table S2. Top 10 differentially downregulated and upregulated metabolites.


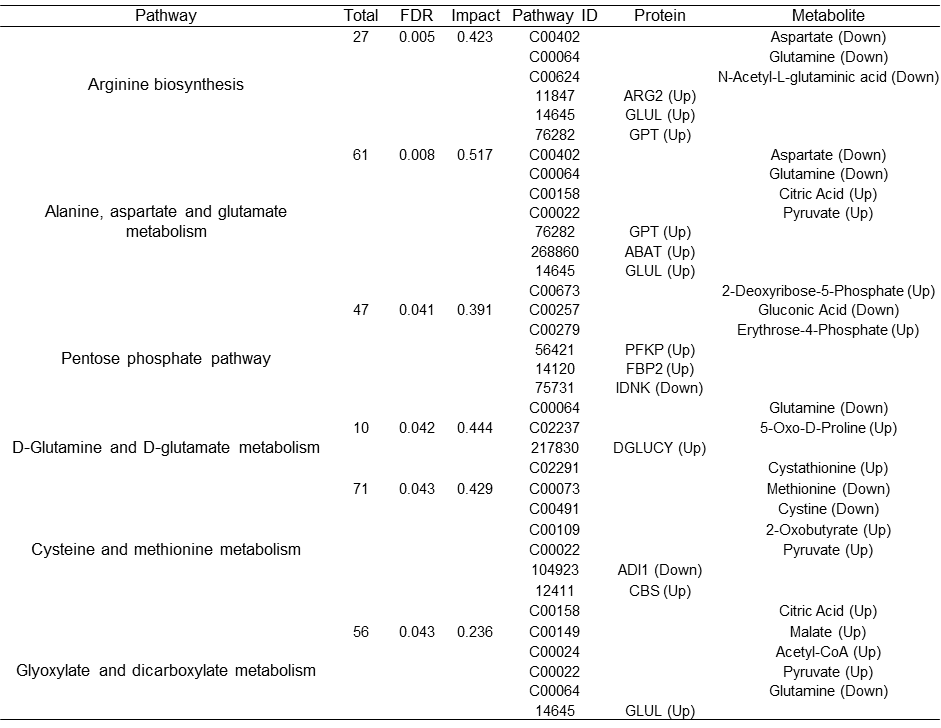


Table S3. Significant 6 differential pathways including proteins and metabolites.


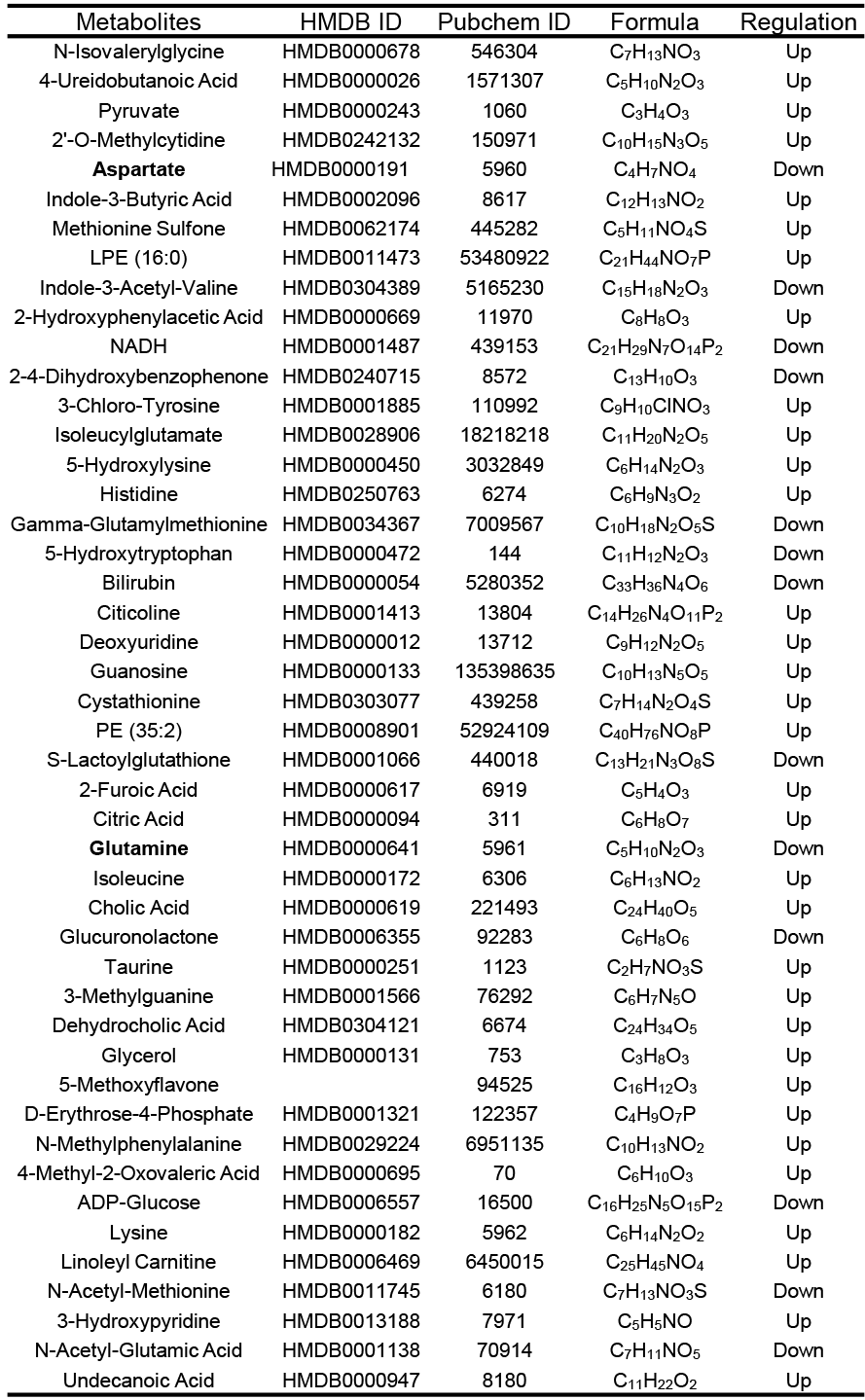


**Table S4.** Core metabolites with a metabolite-protein-islet function indicators degree > 25.


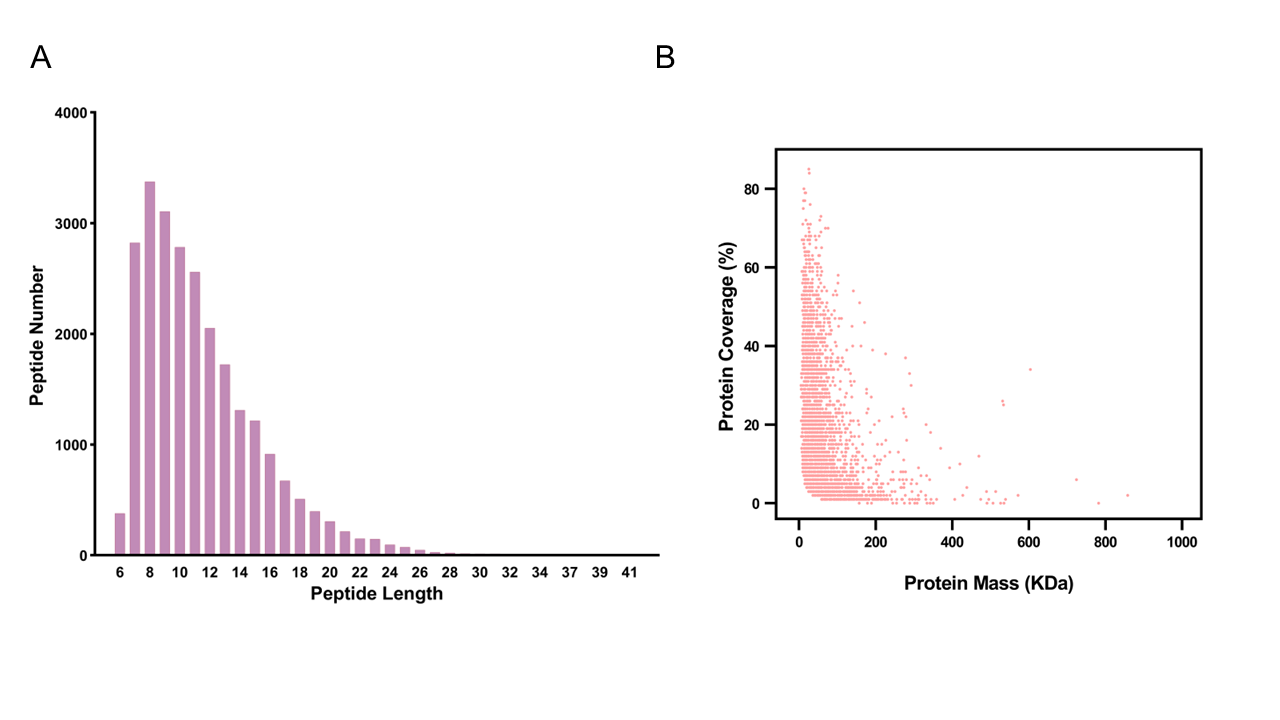
**Figure S1.** Features of the quantified peptides and proteins. (A) Distribution map of identified peptide lengths. (B) Molecular weight and coverage distribution map of the identified proteins.


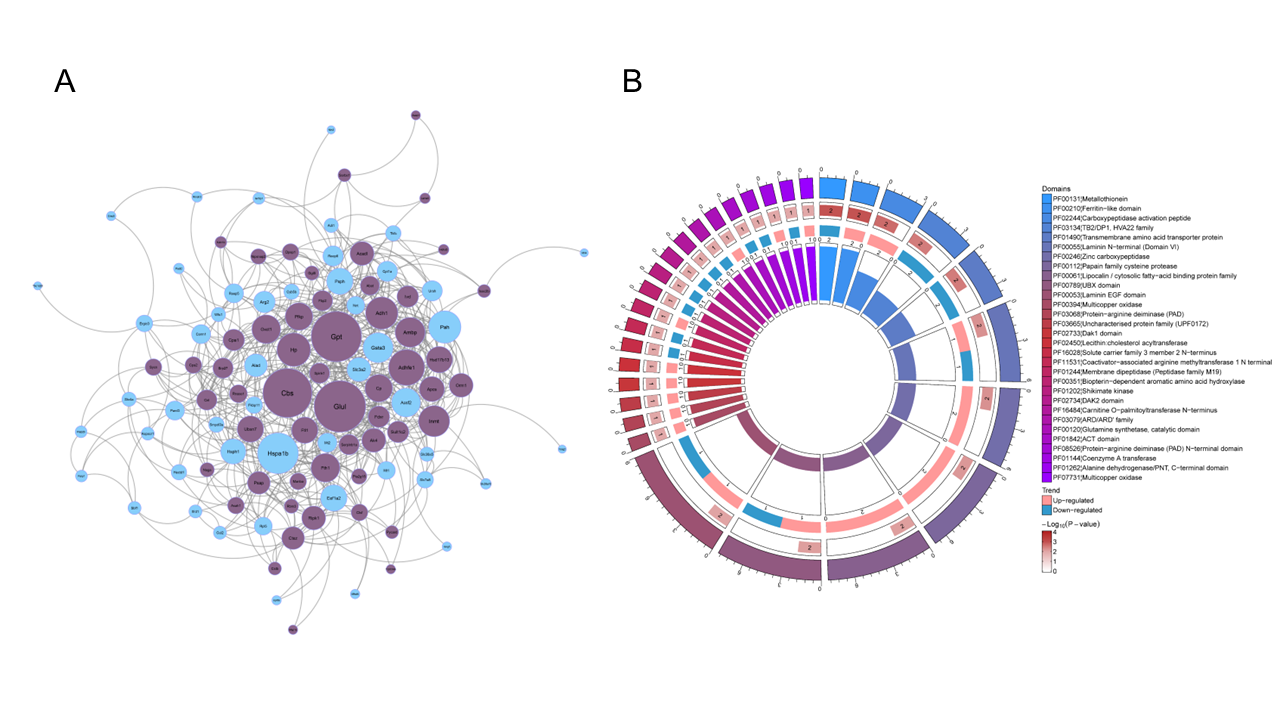


**Figure S2.** (A) The protein‒protein interaction network of the differentially expressed proteins. The node size corresponds to the node degree, and the color indicates the fold change, with purple representing upregulated proteins and blue representing downregulated proteins. (B) The top 30 domain enrichment analyses of the differentially expressed proteins.

**
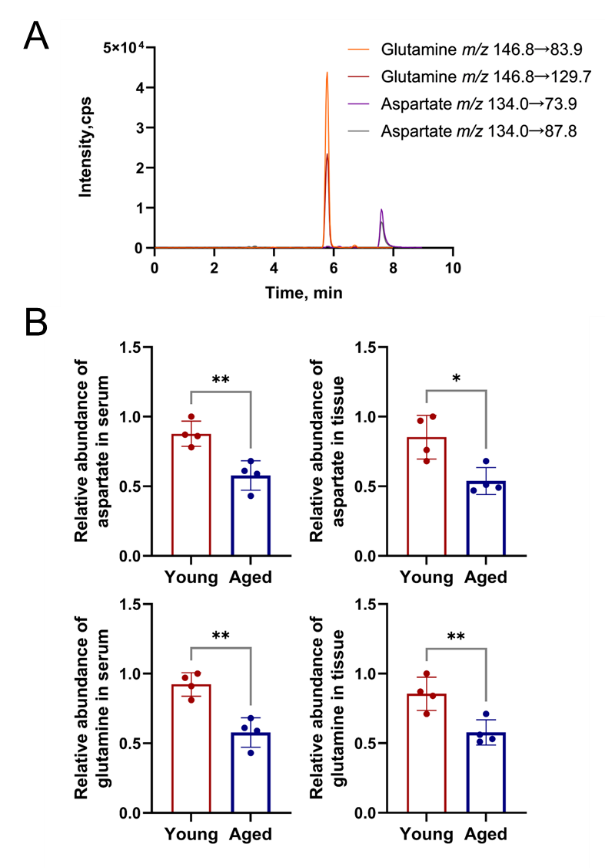
**

**Figure S3.** (A) A typical MRM chromatogram of target metabolites. (B) Respective abundance of aspartate and glutamine in serum and tissue.

**Method S1.** Multiple reaction monitoring (MRM)-based aspartate and glutamine quantification

Plasma was immediately separated from whole blood samples that were collected with EDTA vacuum tubes (BD Vacutainer, Franklin Lakes, NJ, USA) and then stored at -80°C. 100 µL of each plasma sample was slowly lysed, four volumes (400 µL) of precooled methanol were added, and the mixture was vortexed and stored at -80°C for 8 h to allow protein precipitation by centrifugation at 14,000 rpm for 10 min at 4°C. The supernatant was collected, evaporated, reconstituted and used for analysis. For absolute quantitative analysis of potential metabolic markers in the cohort, a SCIEX Exion LC AD system (AB SCIEX, MA, USA) and a QTRAP 6500 mass spectrometer (AB SCIEX, MA, USA) were employed for MRM-based target metabolite quantification. The sample preparation and analysis were performed in basically the same way as in the protocol described previously. The metabolites were separated at a flow rate of 0.3 mL/min with a 10 min gradient (buffer B, 10% (0 min) → 10% (1 min) → 90% (4 min) → 90% (8 min) → 10% (9 min)) using an analytical column (2.7 μm, 30 mm × 3.1 mm; Agilent Technologies, USA), and Q1 and Q3 were both set at unit resolution. At least two product ions became a unique signature in combination with the precursor ion for MRM analysis, and MRM transitions of m/z 146.8→83.9 and m/z 146.8→129.7 for glutamine, m/z 134.0→73.9 and m/z 134.0→87.8 for Aspartate were established, which could then be specific target metabolites for quantitation purposes. Data were collected and analyzed using AB SCIEX Analyst software, sample concentration was quantitated by summation of transitions, and the average metabolite standard intensity was acquired and used to represent the intensity of the target metabolite.
